# *N*-[(1*E*)-5-(3-Chloro­phen­yl)-3-methyl­cyclo­hex-2-en-1-yl­idene]hydroxyl­amine

**DOI:** 10.1107/S1600536813004698

**Published:** 2013-02-28

**Authors:** G. Ganesh, K. Murugavel, P. S. Kannan, S. Amirthaganesan, A. SubbiahPandi

**Affiliations:** a Department of Physics, S.M.K. Fomra Institute of Technology, Thaiyur, Chennai 603 103, India; b Department of Chemistry, Saveetha Engineering College, Chennai, India; c Department of Physics, Presidency College (Autonomous), Chennai 600 005, India

## Abstract

The whole of the title mol­ecule, C_13_H_14_ClNO, is disordered over two sets of sites with a refined occupancy ratio of 0.560 (6):0.440 (6). The oxime group having a C=N double bond adopts an *E* conformation. The dihedral angles between the rings (all atoms) are 89.5 (5) (major componenent) and 88.0 (6)° (minor component).

## Related literature

For applications of oximes, see: Kukushkin *et al.* (1996 ▶): Chaudhuri (2003 ▶). For a related structure of a chloro­phenyl oxime derivative, see: Ravichandran *et al.* (2010 ▶).
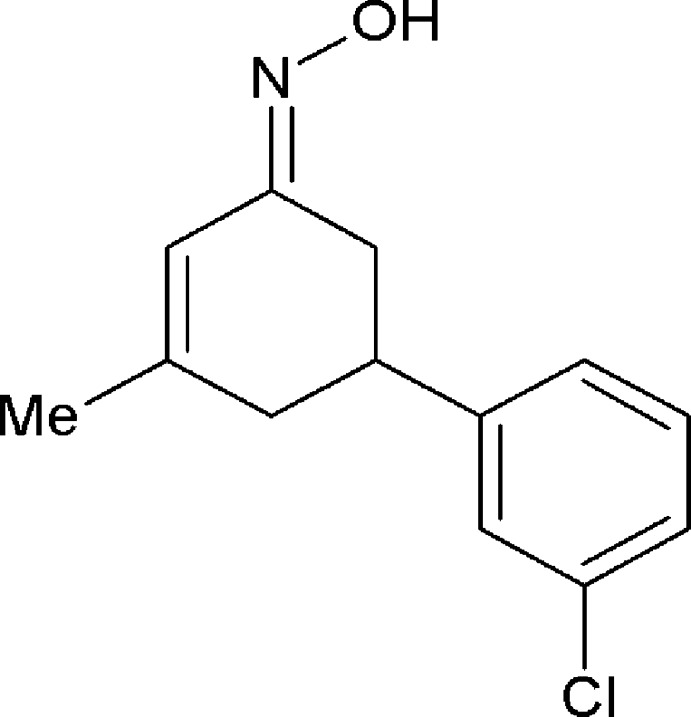


## Experimental

### 

#### Crystal data

C_13_H_14_ClNO
*M* *_r_* = 235.70
Tetragonal, 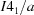

*a* = 19.7898 (8) Å
*c* = 12.4416 (11) Å
*V* = 4872.6 (5) Å^3^
*Z* = 16
Mo *K*α radiation
μ = 0.29 mm^−1^
*T* = 295 K
0.25 × 0.22 × 0.19 mm

#### Data collection

Bruker APEXII CCD area-detector diffractometer
Absorption correction: multi-scan (*SADABS*; Sheldrick, 1996 ▶) *T*_min_ = 0.930, *T*_max_ = 0.946
19841 measured reflections
2394 independent reflections
1765 reflections with *I* > 2σ(*I*)
*R*_int_ = 0.034

#### Refinement

*R*[*F* ^2^ > 2σ(*F* ^2^)] = 0.080
*wR*(*F* ^2^) = 0.242
*S* = 1.05
2394 reflections
255 parameters
93 restraints
H-atom parameters constrained
Δρ_max_ = 0.72 e Å^−3^
Δρ_min_ = −0.50 e Å^−3^

### 

Data collection: *APEX2* (Bruker, 2008 ▶); cell refinement: *SAINT* (Bruker, 2008 ▶); data reduction: *SAINT*; program(s) used to solve structure: *SHELXS97* (Sheldrick, 2008 ▶); program(s) used to refine structure: *SHELXL97* (Sheldrick, 2008 ▶); molecular graphics: *ORTEP-3 for Windows* (Farrugia, 2012 ▶); software used to prepare material for publication: *SHELXL97* and *PLATON* (Spek, 2009 ▶).

## Supplementary Material

Crystal structure: contains datablock(s) global, I. DOI: 10.1107/S1600536813004698/nk2191sup1.cif

Structure factors: contains datablock(s) I. DOI: 10.1107/S1600536813004698/nk2191Isup2.hkl

Supplementary material file. DOI: 10.1107/S1600536813004698/nk2191Isup3.cml

Additional supplementary materials:  crystallographic information; 3D view; checkCIF report

## Figures and Tables

**Fig. 1.**
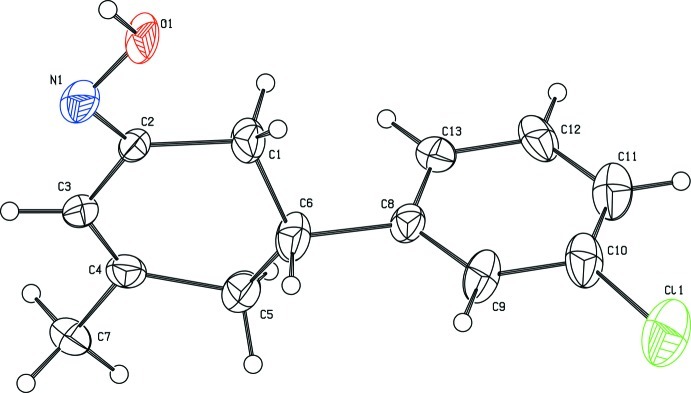
The molecular structure of the major component of title compound with 30% probability displacement ellipsoids.

**Fig. 2.**
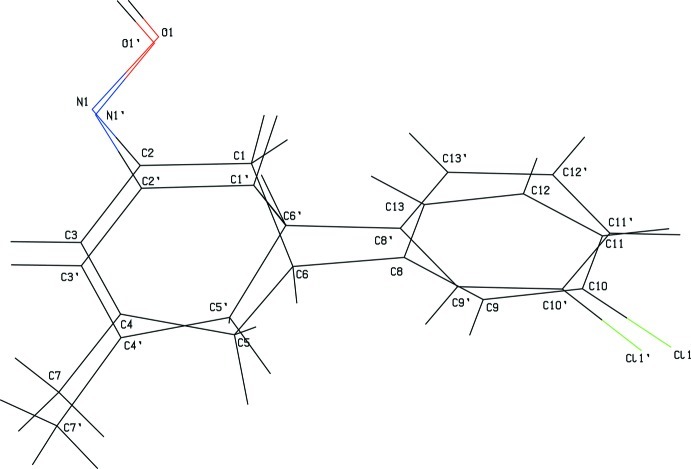
Stick plot of both major and minor components of the title compound with atom labels for non-H atoms.

## supplementary crystallographic information

### Comment

Oximes are a classical type of chelating ligands which are widely used in coordination and analytical chemistry (Kukushkin *et al.*, 1996; Chaudhuri, 2003).

The bond lengths and bond angles of the title compound are comparable with those observed in other chlorophenyl oxime derivative. (Ravichandran *et al.*, 2010). The whole molecule of the title compound is disordered over two positions (Fig. 1 and 2) with a refined occupancy ratio of 0.560:0.440 (6). For the major disorder componenent, the phenyl ring [C8—C13] is almost perpendicular to the cyclohexene ring [C1—C6] with dihedral angle 89.5 (5) °. Similarly, for the minor disorder component, the phenyl ring [C8'-C13'] is almost perpendicular to the cyclohexene ring [C1'-C6'] with dihedral angle 88.0 (6) °. In the crystal unit, the oxime groups C2=N1 and C2'=N1' double bond exists in E configuration, which is confirmed by the torsion angle of -177.7 (2) and 177.0 (2)° of C3—C2—N1—O1 and C3'-C2'-N1'-O1' moeity respectively. Phenyl ring is completely planar, while cyclohexene ring is far from being planar with maximum deviations from the mean plane being 0.195 (2) and 0.241 (7) Å for the C1 and C6' atoms respectively.

### Experimental

To a stirred suspension of benzo[*c*]furan (2.38 g, 7.437 mmol) in dry THF (20 ml), lead tetraacetate (3.2 g, 7.437 mmol) was added and refluxed at 50°C for half an hour. The reaction mixture was then poured into water (200 ml) and extracted with ethyl acetate (2 *x* 20 ml), washed with brine solution and dried (Na_2_SO_4_). The removal of solvent *in vacuo* followed by crystallization from methanol afforded the title compound as a colorless solid. Single crystals suitable for X-ray diffraction were obtained by slow evaporation of a solution of the title compound in methanol at room temperature.

### Refinement

The whole molecule was disordered over two sites (C1—C13/Cl/N1/O1 and C1'-C13'/Cl'/N1'/O1') with refined occupancy ratio 0.560:0.440 (6). Displacement ellipsoid and bond distance similarity restraints were employed to control the geometry of both disorder components. The residual factors are R1 = 0.0803 and wR2 = 0.2417 are rather high. The data were collected with the body centred tetragonal cell as the Bravais cell and processed in the Laue class 4/m. The multiscan absorption correction was done assuming in the crystal to be medium absorber. The residual factors are high, probably due to the restraints needed to account for completely disordered molecule. The actual disorder probably could be several fold with each having slightly different orientation. The idealized two fold disorder may not be accounting for the full disorder. All H atoms were fixed geometrically and allowed to ride on their parent C atoms, with C—H distances fixed in the range 0.93–0.97 Å with *U*_iso_(H) = 1.2*U*_eq_(C) for other H atoms.

### Crystal data

**Table:**

C_13_H_14_ClNO	*D*_x_ = 1.285 Mg m^−^^3^
*M**_r_* = 235.70	Mo *K*α radiation, λ = 0.71073 Å
Tetragonal, *I*4_1_/*a*	Cell parameters from 2394 reflections
Hall symbol: -I 4ad	θ = 1.5–26.0°
*a* = 19.7898 (8) Å	µ = 0.29 mm^−^^1^
*c* = 12.4416 (11) Å	*T* = 295 K
*V* = 4872.6 (5) Å^3^	Block, white
*Z* = 16	0.25 × 0.22 × 0.19 mm
*F*(000) = 1984	

### Data collection

**Table:**

Bruker APEXII CCD area-detector diffractometer	2394 independent reflections
Radiation source: fine-focus sealed tube	1765 reflections with *I* > 2σ(*I*)
Graphite monochromator	*R*_int_ = 0.034
ω and φ scans	θ_max_ = 26.0°, θ_min_ = 1.5°
Absorption correction: multi-scan (*SADABS*; Sheldrick, 1996)	*h* = −24→24
*T*_min_ = 0.930, *T*_max_ = 0.946	*k* = −24→24
19841 measured reflections	*l* = −15→15

### Refinement

**Table:**

Refinement on *F*^2^	Primary atom site location: structure-invariant direct methods
Least-squares matrix: full	Secondary atom site location: difference Fourier map
*R*[*F*^2^ > 2σ(*F*^2^)] = 0.080	Hydrogen site location: inferred from neighbouring sites
*wR*(*F*^2^) = 0.242	H-atom parameters constrained
*S* = 1.05	*w* = 1/[σ^2^(*F*_o_^2^) + (0.1164*P*)^2^ + 10.7128*P*] where *P* = (*F*_o_^2^ + 2*F*_c_^2^)/3
2394 reflections	(Δ/σ)_max_ = 0.026
255 parameters	Δρ_max_ = 0.72 e Å^−^^3^
93 restraints	Δρ_min_ = −0.50 e Å^−^^3^

### Special details

**Table:**

Geometry. All e.s.d.'s (except the e.s.d. in the dihedral angle between two l.s. planes) are estimated using the full covariance matrix. The cell e.s.d.'s are taken into account individually in the estimation of e.s.d.'s in distances, angles and torsion angles; correlations between e.s.d.'s in cell parameters are only used when they are defined by crystal symmetry. An approximate (isotropic) treatment of cell e.s.d.'s is used for estimating e.s.d.'s involving l.s. planes.
Refinement. Refinement of *F*^2^ against ALL reflections. The weighted *R*-factor *wR* and goodness of fit *S* are based on *F*^2^, conventional *R*-factors *R* are based on *F*, with *F* set to zero for negative *F*^2^. The threshold expression of *F*^2^ > σ(*F*^2^) is used only for calculating *R*-factors(gt) *etc*. and is not relevant to the choice of reflections for refinement. *R*-factors based on *F*^2^ are statistically about twice as large as those based on *F*, and *R*- factors based on ALL data will be even larger.

### Fractional atomic coordinates and isotropic or equivalent isotropic displacement parameters (Å^2^)

**Table:**

	*x*	*y*	*z*	*U*_iso_*/*U*_eq_	Occ. (<1)
O1	0.5714 (9)	0.6964 (10)	0.5126 (13)	0.064 (4)	0.560 (6)
H1	0.5873	0.7176	0.5632	0.095*	0.560 (6)
N1	0.5172 (8)	0.6572 (8)	0.5484 (11)	0.055 (4)	0.560 (6)
C1	0.5202 (8)	0.6216 (9)	0.3560 (9)	0.053 (4)	0.560 (6)
H1A	0.5687	0.6283	0.3589	0.064*	0.560 (6)
H1B	0.5010	0.6582	0.3141	0.064*	0.560 (6)
C2	0.4922 (8)	0.6246 (8)	0.4677 (7)	0.036 (3)	0.560 (6)
C3	0.4336 (8)	0.5833 (7)	0.4904 (12)	0.044 (4)	0.560 (6)
H3	0.4135	0.5871	0.5577	0.053*	0.560 (6)
C4	0.4070 (6)	0.5404 (6)	0.4205 (13)	0.043 (3)	0.560 (6)
C5	0.4409 (10)	0.5208 (11)	0.3142 (15)	0.055 (5)	0.560 (6)
H5A	0.4480	0.4723	0.3128	0.067*	0.560 (6)
H5B	0.4111	0.5323	0.2550	0.067*	0.560 (6)
C6	0.5054 (4)	0.5550 (4)	0.2999 (5)	0.0570 (18)	0.560 (6)
H6	0.5377	0.5238	0.3331	0.068*	0.560 (6)
C7	0.34307 (17)	0.49939 (17)	0.4407 (3)	0.057 (2)	0.560 (6)
H7A	0.3338	0.4715	0.3793	0.085*	0.560 (6)
H7B	0.3058	0.5295	0.4527	0.085*	0.560 (6)
H7C	0.3494	0.4713	0.5028	0.085*	0.560 (6)
C8	0.52861 (17)	0.55769 (17)	0.1822 (3)	0.0421 (17)	0.560 (6)
C9	0.58200 (17)	0.51926 (17)	0.1431 (3)	0.0541 (19)	0.560 (6)
H9	0.6054	0.4904	0.1888	0.065*	0.560 (6)
C10	0.60045 (17)	0.52400 (17)	0.0355 (3)	0.063 (3)	0.560 (6)
C11	0.56550 (17)	0.56717 (17)	−0.0328 (3)	0.066 (3)	0.560 (6)
H11	0.5778	0.5703	−0.1048	0.080*	0.560 (6)
C12	0.51211 (17)	0.60561 (17)	0.0063 (3)	0.057 (2)	0.560 (6)
H12	0.4887	0.6345	−0.0395	0.069*	0.560 (6)
C13	0.49367 (17)	0.60087 (17)	0.1138 (3)	0.055 (2)	0.560 (6)
H13	0.4580	0.6266	0.1400	0.066*	0.560 (6)
Cl1	0.6641 (5)	0.4726 (6)	−0.0028 (7)	0.105 (2)	0.560 (6)
O1'	0.5722 (9)	0.6919 (14)	0.5195 (19)	0.072 (7)	0.440 (6)
H1'	0.5786	0.7190	0.5682	0.108*	0.440 (6)
N1'	0.5138 (8)	0.6543 (11)	0.5411 (14)	0.049 (5)	0.440 (6)
C1'	0.5283 (9)	0.6052 (12)	0.3642 (11)	0.054 (5)	0.440 (6)
H1'1	0.5638	0.5713	0.3650	0.064*	0.440 (6)
H1'2	0.5491	0.6484	0.3485	0.064*	0.440 (6)
C2'	0.4964 (11)	0.6083 (10)	0.4731 (9)	0.039 (4)	0.440 (6)
C3'	0.4381 (9)	0.5666 (9)	0.4972 (14)	0.036 (3)	0.440 (6)
H3'	0.4183	0.5704	0.5647	0.043*	0.440 (6)
C4'	0.4117 (7)	0.5234 (8)	0.4276 (16)	0.040 (3)	0.440 (6)
C5'	0.4337 (12)	0.5340 (13)	0.3107 (17)	0.049 (4)	0.440 (6)
H5'1	0.4548	0.4921	0.2880	0.059*	0.440 (6)
H5'2	0.3926	0.5384	0.2689	0.059*	0.440 (6)
C6'	0.4783 (3)	0.5882 (4)	0.2756 (5)	0.0406 (16)	0.440 (6)
H6'	0.4491	0.6280	0.2699	0.049*	0.440 (6)
C7'	0.3566 (2)	0.4738 (2)	0.4612 (4)	0.052 (2)	0.440 (6)
H7'1	0.3422	0.4482	0.3997	0.078*	0.440 (6)
H7'2	0.3188	0.4983	0.4900	0.078*	0.440 (6)
H7'3	0.3740	0.4437	0.5149	0.078*	0.440 (6)
C8'	0.5110 (2)	0.5810 (2)	0.1653 (4)	0.039 (2)	0.440 (6)
C9'	0.5606 (2)	0.5320 (2)	0.1517 (4)	0.0379 (19)	0.440 (6)
H9'	0.5722	0.5039	0.2086	0.046*	0.440 (6)
C10'	0.5928 (2)	0.5251 (2)	0.0530 (4)	0.045 (2)	0.440 (6)
C11'	0.5754 (2)	0.5671 (2)	−0.0321 (4)	0.052 (3)	0.440 (6)
H11'	0.5969	0.5625	−0.0981	0.063*	0.440 (6)
C12'	0.5258 (2)	0.6161 (2)	−0.0185 (4)	0.058 (3)	0.440 (6)
H12'	0.5141	0.6443	−0.0755	0.070*	0.440 (6)
C13'	0.4936 (2)	0.6231 (2)	0.0802 (4)	0.049 (2)	0.440 (6)
H13'	0.4604	0.6558	0.0892	0.059*	0.440 (6)
Cl1'	0.6585 (5)	0.4711 (6)	0.0291 (8)	0.0866 (18)	0.440 (6)

### Atomic displacement parameters (Å^2^)

**Table:**

	*U*^11^	*U*^22^	*U*^33^	*U*^12^	*U*^13^	*U*^23^
O1	0.087 (9)	0.075 (7)	0.028 (5)	−0.022 (6)	0.006 (5)	−0.025 (4)
N1	0.080 (9)	0.053 (7)	0.033 (5)	0.002 (5)	−0.007 (5)	−0.011 (5)
C1	0.067 (6)	0.060 (8)	0.033 (4)	−0.015 (5)	0.006 (3)	−0.010 (4)
C2	0.046 (4)	0.032 (7)	0.032 (3)	0.008 (4)	0.004 (3)	−0.003 (3)
C3	0.059 (5)	0.032 (7)	0.042 (4)	0.010 (5)	0.008 (3)	0.002 (4)
C4	0.046 (4)	0.034 (7)	0.051 (4)	0.004 (4)	0.002 (3)	0.007 (4)
C5	0.065 (7)	0.050 (6)	0.052 (6)	−0.004 (6)	0.010 (5)	−0.012 (4)
C6	0.058 (4)	0.073 (4)	0.040 (3)	−0.009 (3)	0.007 (3)	−0.018 (3)
C7	0.067 (4)	0.044 (4)	0.060 (4)	−0.005 (3)	0.015 (3)	0.011 (3)
C8	0.048 (4)	0.044 (4)	0.035 (3)	−0.004 (3)	0.000 (3)	−0.010 (3)
C9	0.019 (3)	0.082 (5)	0.061 (4)	0.000 (3)	0.009 (2)	−0.021 (3)
C10	0.044 (4)	0.090 (7)	0.057 (5)	−0.020 (4)	0.015 (4)	−0.011 (4)
C11	0.052 (4)	0.097 (10)	0.050 (6)	−0.020 (4)	0.003 (4)	−0.017 (5)
C12	0.050 (4)	0.067 (4)	0.055 (4)	−0.022 (3)	0.011 (3)	0.010 (3)
C13	0.065 (4)	0.038 (4)	0.063 (5)	−0.003 (3)	0.019 (4)	0.003 (4)
Cl1	0.073 (2)	0.133 (3)	0.107 (5)	0.0165 (18)	0.030 (3)	−0.023 (3)
O1'	0.051 (8)	0.104 (13)	0.061 (11)	−0.032 (8)	0.006 (7)	−0.015 (8)
N1'	0.034 (6)	0.074 (11)	0.041 (8)	−0.008 (6)	0.017 (5)	−0.014 (7)
C1'	0.060 (7)	0.062 (11)	0.039 (5)	−0.023 (7)	0.007 (4)	−0.006 (5)
C2'	0.054 (6)	0.026 (8)	0.036 (4)	0.009 (6)	0.001 (4)	−0.002 (4)
C3'	0.041 (4)	0.027 (8)	0.038 (4)	0.014 (4)	0.008 (3)	0.007 (4)
C4'	0.049 (5)	0.021 (7)	0.049 (5)	0.011 (4)	0.003 (4)	0.003 (5)
C5'	0.049 (5)	0.054 (10)	0.043 (5)	−0.003 (6)	−0.006 (4)	−0.013 (5)
C6'	0.040 (3)	0.045 (4)	0.037 (3)	0.016 (3)	0.000 (3)	−0.005 (3)
C7'	0.040 (4)	0.057 (5)	0.058 (5)	−0.005 (3)	−0.004 (3)	0.017 (4)
C8'	0.045 (4)	0.030 (5)	0.041 (4)	0.012 (3)	−0.005 (3)	0.003 (3)
C9'	0.016 (4)	0.062 (5)	0.035 (4)	0.011 (3)	0.009 (3)	−0.009 (3)
C10'	0.042 (5)	0.065 (6)	0.030 (4)	−0.003 (4)	0.010 (4)	−0.023 (3)
C11'	0.053 (5)	0.065 (8)	0.040 (6)	−0.015 (4)	0.011 (4)	−0.015 (5)
C12'	0.048 (5)	0.076 (6)	0.051 (5)	−0.015 (4)	0.023 (4)	0.007 (4)
C13'	0.063 (5)	0.049 (5)	0.035 (4)	0.002 (4)	0.016 (3)	0.010 (3)
Cl1'	0.065 (3)	0.099 (3)	0.096 (5)	0.0217 (19)	0.016 (3)	−0.007 (3)

### Geometric parameters (Å, º)

**Table:**

O1—N1	1.396 (7)	O1'—N1'	1.400 (7)
O1—H1	0.8200	O1'—H1'	0.8200
N1—C2	1.292 (8)	N1'—C2'	1.290 (9)
C1—C2	1.497 (7)	C1'—C2'	1.496 (8)
C1—C6	1.520 (12)	C1'—C6'	1.520 (13)
C1—H1A	0.9700	C1'—H1'1	0.9700
C1—H1B	0.9700	C1'—H1'2	0.9700
C2—C3	1.448 (7)	C2'—C3'	1.449 (8)
C3—C4	1.324 (7)	C3'—C4'	1.324 (8)
C3—H3	0.9300	C3'—H3'	0.9300
C4—C7	1.524 (6)	C4'—C7'	1.525 (7)
C4—C5	1.532 (10)	C4'—C5'	1.533 (10)
C5—C6	1.456 (10)	C5'—C6'	1.454 (10)
C5—H5A	0.9700	C5'—H5'1	0.9700
C5—H5B	0.9700	C5'—H5'2	0.9700
C6—C8	1.535 (6)	C6'—C8'	1.525 (6)
C6—H6	0.9800	C6'—H6'	0.9800
C7—H7A	0.9600	C7'—H7'1	0.9600
C7—H7B	0.9600	C7'—H7'2	0.9600
C7—H7C	0.9600	C7'—H7'3	0.9600
C8—C9	1.3900	C8'—C13'	1.3900
C8—C13	1.3900	C8'—C9'	1.3900
C9—C10	1.3900	C9'—C10'	1.3900
C9—H9	0.9300	C9'—H9'	0.9300
C10—C11	1.3900	C10'—C11'	1.3900
C10—Cl1	1.687 (5)	C10'—Cl1'	1.710 (6)
C11—C12	1.3900	C11'—C12'	1.3900
C11—H11	0.9300	C11'—H11'	0.9300
C12—C13	1.3900	C12'—C13'	1.3900
C12—H12	0.9300	C12'—H12'	0.9300
C13—H13	0.9300	C13'—H13'	0.9300
			
N1—O1—H1	109.5	N1'—O1'—H1'	109.5
C2—N1—O1	108.8 (13)	C2'—N1'—O1'	118.0 (16)
C2—C1—C6	112.9 (9)	C2'—C1'—C6'	113.0 (10)
C2—C1—H1A	109.0	C2'—C1'—H1'1	109.0
C6—C1—H1A	109.0	C6'—C1'—H1'1	109.0
C2—C1—H1B	109.0	C2'—C1'—H1'2	109.0
C6—C1—H1B	109.0	C6'—C1'—H1'2	109.0
H1A—C1—H1B	107.8	H1'1—C1'—H1'2	107.8
N1—C2—C3	115.9 (11)	N1'—C2'—C3'	118.6 (13)
N1—C2—C1	126.9 (11)	N1'—C2'—C1'	120.7 (13)
C3—C2—C1	117.1 (7)	C3'—C2'—C1'	120.0 (9)
C4—C3—C2	123.5 (9)	C4'—C3'—C2'	123.1 (10)
C4—C3—H3	118.2	C4'—C3'—H3'	118.5
C2—C3—H3	118.2	C2'—C3'—H3'	118.5
C3—C4—C7	124.2 (11)	C3'—C4'—C7'	121.2 (13)
C3—C4—C5	123.7 (9)	C3'—C4'—C5'	114.8 (10)
C7—C4—C5	111.8 (7)	C7'—C4'—C5'	123.4 (10)
C6—C5—C4	111.8 (9)	C6'—C5'—C4'	123.9 (11)
C6—C5—H5A	109.3	C6'—C5'—H5'1	106.4
C4—C5—H5A	109.3	C4'—C5'—H5'1	106.4
C6—C5—H5B	109.3	C6'—C5'—H5'2	106.4
C4—C5—H5B	109.3	C4'—C5'—H5'2	106.4
H5A—C5—H5B	107.9	H5'1—C5'—H5'2	106.4
C5—C6—C1	121.1 (10)	C5'—C6'—C1'	109.9 (12)
C5—C6—C8	113.3 (8)	C5'—C6'—C8'	117.3 (9)
C1—C6—C8	110.5 (7)	C1'—C6'—C8'	113.4 (7)
C5—C6—H6	103.2	C5'—C6'—H6'	105.0
C1—C6—H6	103.2	C1'—C6'—H6'	105.0
C8—C6—H6	103.2	C8'—C6'—H6'	105.0
C4—C7—H7A	109.5	C4'—C7'—H7'1	109.5
C4—C7—H7B	109.5	C4'—C7'—H7'2	109.5
H7A—C7—H7B	109.5	H7'1—C7'—H7'2	109.5
C4—C7—H7C	109.5	C4'—C7'—H7'3	109.5
H7A—C7—H7C	109.5	H7'1—C7'—H7'3	109.5
H7B—C7—H7C	109.5	H7'2—C7'—H7'3	109.5
C9—C8—C13	120.0	C13'—C8'—C9'	120.0
C9—C8—C6	122.9 (4)	C13'—C8'—C6'	121.7 (3)
C13—C8—C6	117.1 (4)	C9'—C8'—C6'	118.3 (3)
C8—C9—C10	120.0	C10'—C9'—C8'	120.0
C8—C9—H9	120.0	C10'—C9'—H9'	120.0
C10—C9—H9	120.0	C8'—C9'—H9'	120.0
C9—C10—C11	120.0	C11'—C10'—C9'	120.0
C9—C10—Cl1	115.3 (3)	C11'—C10'—Cl1'	115.5 (4)
C11—C10—Cl1	124.6 (3)	C9'—C10'—Cl1'	124.4 (4)
C12—C11—C10	120.0	C10'—C11'—C12'	120.0
C12—C11—H11	120.0	C10'—C11'—H11'	120.0
C10—C11—H11	120.0	C12'—C11'—H11'	120.0
C11—C12—C13	120.0	C13'—C12'—C11'	120.0
C11—C12—H12	120.0	C13'—C12'—H12'	120.0
C13—C12—H12	120.0	C11'—C12'—H12'	120.0
C12—C13—C8	120.0	C12'—C13'—C8'	120.0
C12—C13—H13	120.0	C12'—C13'—H13'	120.0
C8—C13—H13	120.0	C8'—C13'—H13'	120.0
			
O1—N1—C2—C3	−177.7 (17)	O1'—N1'—C2'—C3'	177 (2)
O1—N1—C2—C1	7 (3)	O1'—N1'—C2'—C1'	−13 (4)
C6—C1—C2—N1	149 (2)	C6'—C1'—C2'—N1'	−140 (2)
C6—C1—C2—C3	−27 (2)	C6'—C1'—C2'—C3'	30 (3)
N1—C2—C3—C4	−171.4 (18)	N1'—C2'—C3'—C4'	172 (2)
C1—C2—C3—C4	5 (3)	C1'—C2'—C3'—C4'	2 (3)
C2—C3—C4—C7	−176.5 (15)	C2'—C3'—C4'—C7'	171.5 (18)
C2—C3—C4—C5	10 (3)	C2'—C3'—C4'—C5'	−16 (3)
C3—C4—C5—C6	0 (3)	C3'—C4'—C5'—C6'	−2 (4)
C7—C4—C5—C6	−174.3 (14)	C7'—C4'—C5'—C6'	170.0 (19)
C4—C5—C6—C1	−25 (3)	C4'—C5'—C6'—C1'	32 (3)
C4—C5—C6—C8	−159.4 (14)	C4'—C5'—C6'—C8'	163 (2)
C2—C1—C6—C5	38 (2)	C2'—C1'—C6'—C5'	−43 (2)
C2—C1—C6—C8	174.1 (12)	C2'—C1'—C6'—C8'	−176.8 (15)
C5—C6—C8—C9	−108.1 (13)	C5'—C6'—C8'—C13'	111.6 (16)
C1—C6—C8—C9	112.4 (10)	C1'—C6'—C8'—C13'	−118.5 (12)
C5—C6—C8—C13	71.8 (13)	C5'—C6'—C8'—C9'	−70.0 (16)
C1—C6—C8—C13	−67.7 (10)	C1'—C6'—C8'—C9'	59.9 (12)
C13—C8—C9—C10	0.0	C13'—C8'—C9'—C10'	0.0
C6—C8—C9—C10	179.9 (3)	C6'—C8'—C9'—C10'	−178.4 (4)
C8—C9—C10—C11	0.0	C8'—C9'—C10'—C11'	0.0
C8—C9—C10—Cl1	−177.8 (5)	C8'—C9'—C10'—Cl1'	175.1 (6)
C9—C10—C11—C12	0.0	C9'—C10'—C11'—C12'	0.0
Cl1—C10—C11—C12	177.6 (6)	Cl1'—C10'—C11'—C12'	−175.5 (6)
C10—C11—C12—C13	0.0	C10'—C11'—C12'—C13'	0.0
C11—C12—C13—C8	0.0	C11'—C12'—C13'—C8'	0.0
C9—C8—C13—C12	0.0	C9'—C8'—C13'—C12'	0.0
C6—C8—C13—C12	−179.9 (3)	C6'—C8'—C13'—C12'	178.4 (4)
